# A qualitative study of the views of patients with long-term conditions on family doctors in Hong Kong

**DOI:** 10.1186/1471-2296-11-46

**Published:** 2010-06-04

**Authors:** Stewart W Mercer, Judy Y Siu, Sheila M Hillier, Cindy LK Lam, Yvonne YC Lo, Tai Pong Lam, Sian M Griffiths

**Affiliations:** 1 Section of General Practice and Primary Care, Division of Community-based Sciences, Faculty of Medicine, University of Glasgow, UK; 2 Department of Community & Family Medicine, School of Public Health and Primary Care, The Chinese University of Hong Kong, Hong Kong; 3 Centre for Health Sciences, Barts and The London School of Medicine and Dentistry, Queen Mary, University of London, London, UK; 4 Family Medicine Unit, Department of Medicine, The University of Hong Kong, Hong Kong

## Abstract

**Background:**

Primary care based management of long-term conditions (LTCs) is high on the international healthcare agenda, including the Asia-Pacific region. Hong Kong has a 'mixed economy' healthcare system with both public and private sectors with a range of types of primary care doctors. Recent Hong Kong Government policy aims to enhance the management of LTCs in primary care possibly based on a 'family doctor' model. Patients' views on this are not well documented and the aim of the present study was to explore the views of patients with LTCs on family doctors in Hong Kong.

**Methods:**

The views of patients (with a variety of LTCs) on family doctors in Hong Kong were explored. Two groups of participants were interviewed; a) those who considered themselves as having a family doctor, b) those who considered themselves as not having a family doctor (either with a regular primary care doctor but not a family doctor or with no regular primary care doctor). In-depth individual semi-structured interviews were carried out with 28 participants (10 with a family doctor, 10 with a regular doctor, and 8 with no regular doctor) and analysed using the constant comparative method.

**Results:**

Participants who did not have a family doctor were familiar with the concept but regarded it as a 'luxury item' for the rich within the private healthcare system. Those with a regular family doctor (all private) regarded having one as important to their and their family's health. Participants in both groups felt that as well as the more usual family medicine specialist or general practitioner, traditional Chinese medicine practitioners also had the potential to be family doctors. However most participants attended the public healthcare system for management of their LTCs whether they had a family doctor or not. Cost, perceived need, quality, trust, and choice were all barriers to the use of family doctors for the management of their LTCs.

**Conclusions:**

Important barriers to the adoption of a 'family doctor' model of management of LTCs exist in Hong Kong. Effective policy implementation seems unlikely unless these complex barriers are addressed.

## Background

Effective primary health care is regarded as essential for a high quality, equitable, and cost-effective health care system [1] and a variety of models of primary care are in place around the world [2]. The rising incidence of long-term conditions globally, linked to the changing demographic profiles (with more people living to a greater age) is an important driver for the development of more effective primary care services [3].

In Hong Kong, like many other Asian countries, primary care is provided by doctors who can either be registered doctors with no postgraduate training at all or have a variety of types of postgraduate training or specialisation. Although vocational training in family medicine with formal examination was introduced more than ten years ago, it is not mandatory. Primary healthcare services are thus provided by a range of practitioners, including doctors of traditional Chinese medicine (TCM), non-family medicine specialists, general practitioners (GPs), and specialist family physicians [4]. There is no quality assurance of private primary care. Furthermore, Hong Kong has a 'mixed-economy' system combining a private healthcare system (where people pay for medical services and are free to choose their own doctors) with a public system which is heavily subsidised by the government and organised by the 'Hospital Authority' (HA). Primary care in the public sector is provided mainly by General Out-Patient Centres (GOPC). The private sector is the major provider of primary healthcare with about 70% of primary care consultations being provided privately [5]. The phenomenon of 'doctor shopping' is widespread in the private sector in Hong Kong [4,6].

In Hong Kong, the recent consultative document "Building a Healthy Tomorrow - A Discussion Paper on the Future Service Delivery Model for our Health Care System" of the Health, Welfare and Food Bureau [5] places a strong emphasis on the need for an effective primary care system especially in chronic disease and preventive care. In the 2008-09 Policy Address, the government proposes further to subsidise chronic disease patients in receiving comprehensive treatment, follow-up and care support from private doctors, and is also planning to introduce basic primary care service models focusing on preventive care and a primary care register based on the family-doctor concept. The family-doctor model, therefore, has been put forward as a solution to the rising demand for health care services from the aging Hong Kong population [5].

The aim of the present study was to explore the incentives and barriers to adopting the family doctor model in Hong Kong from the viewpoint of patients with long-term conditions. Specific objectives were to:

1. Examine their knowledge and understanding of the concept of a 'family doctor '.

2. Explore their views on primary care as it currently exists.

3. Elucidate their attitudes towards the role of different primary care providers.

4. Delineate the incentives and barriers to adopting the family doctor model.

## Methods

The present study adopted qualitative methodology and such an approach is especially useful in elucidating issues of context, depth, detail, and content [7]. The choice of qualitative methods reflected our desire to describe what exists 'out there' in the study sample including motivations, experiences and contexts and the reasons for and associations between them [8].

Qualitative research is usually carried out by one-to-one interviews or by focus groups and both methods were considered. In the context of the present study, which we felt would be likely to touch on sensitive areas such as financial status and personal illness, it was decided that one-to-one interviews were the better choice.

In our study, we broadly defined a primary care doctor as any type of doctor whom the subjects would first consult when they need to and a family doctor as one whom they would consult for all types of health problems. This comprehensiveness can help differentiate a family doctor from other types of providers in such a pluralistic primary care system. The identification of a "family doctor" was based on the perception of the subjects. The concept of family doctor is relatively new to Hong Kong citizens as they are free to change primary care doctors at will and there is no agreed standard on training or qualification for the family doctor.

We gathered views from 28 patients with a range of chronic conditions, who considered themselves to as either having (n = 10) or not having (n = 18) a family doctor at present (see below). As far as possible we tried to sample purposively to ensure a maximum variation in terms of age group, gender, socio-economic status (SES), and type of chronic disease (see Table 1).

**Table 1:** Characteristics of participating patients

Participant code	Income Group	Education	Age	Sex	Chronic disease	Marital Status
With family doctor						
5	5	Tertiary	27	M	R	Married
9	5	Tertiary	56	M	B, D, H, S	Married
11	4	Tertiary	23	F	R	Single
12	4	Tertiary	43	F	B, C	Married
13	4	Secondary	47	F	C, R	Married
14	4	Secondary	29	F	B	Married
7	3	Secondary	47	M	R	Divorced
2	2	Primary	63	F	H	Married
8	retired	Tertiary	80	M	H, S	Married
10	NA	Tertiary	52	M	H	Single
With regular doctor but not family doctor						
20	6	Tertiary	43	M	C	Married
21	6	Tertiary	43	F	B	Married
18	6	Secondary	40	F	B	Married
22	5	Secondary	50	F	E	Married
6	3	Tertiary	33	M	B	Married
16	3	Tertiary	26	M	B, R	Single
1	3	Nil	75	M	D, H	Married
17	2	Primary	70	M	H	Married
19	2	Primary	47	F	B, E	Divorced
15	NA	Primary	60	F	B	Divorced
With no regular doctor						
26	4	Secondary	62	F	B	Married
23	3	Secondary	70	M	H	Widowed
25	3	Secondary	48	F	C, H, S	Divorced
27	3	Primary	47	F	B	Married
4	2	Primary	76	F	H	Widowed
3	2	Nil	78	F	D	Widowed
24	1	Primary	72	F	B	Widowed
28	NA	Secondary	51	M	H, S	Married

Key

income (in HK$): chronic conditions:

6: > 40000 B-musculoskeletal

5: 30000 - 40000 C - heart disease

4: 20000 - 30000 D - diabetes

3: 10000 - 20000 E - hyperthyroidism

2: 5000 - 10000 H - hypertension

1: < 5000 R - respiratory problems

NA: refused to answer S - minor stroke

### Recruitment/Interviews

All interviews were carried out between September 2007 and March 2008. Patients were recruited from two sources. Most informants (22 out of the 28 interviewed) were recruited from wave one of the study SHS-P-10 (service utilisation) conducted by Lam http://www.hku.hk/fmunit/research/current_research.htm. This study collected information by telephone survey of a random sample of adults in Hong Kong (see Additional file 1 for the questionnaire and details of the sampling). Informants were asked if they had a regular primary care doctor or not; if yes, they were asked whether they considered this regular doctor to be a family doctor (see additional file 1). Demographic and socio-economic details and also details of chronic diseases of the informants were also gathered. Patients with chronic conditions who were willing to be contacted further were telephoned by the research interviewer and asked to participate in one-to-one interviews. This approach resulted in 22 respondents who were interviewed individually. A weakness of this sampling source was that it was not possible to determine the qualifications of the doctors regarded as 'family doctors'. Thus we supplemented the 'family doctor' group with an additional 6 interviews with chronic disease patients attending a fully qualified family specialist (a Fellow of the Hong Kong College of Family Physicians and a Member of the Hong Kong Academy of Medicine). In this way we were able to discern if the views expressed by those who considered themselves as having a family doctor (of unknown qualification) were similar or different to those attending a known, fully qualified family specialist. Suitable patients (i.e. those with different chronic conditions, ages, and genders) were identified by the practice nurse from patients attending that day, and asked if they would be willing to take part in an interview. They were then introduced to the researcher who was in the practice at that time, and who then carried out the interviews in a quiet room within the practice.

Our final sample was thus as follows:

1. Chronic disease patients with a family doctor at present (n = 10)

2. Chronic disease patients without a family doctor at present;

2.1. With a regular doctor (n = 10)

2.2. With no regular doctor (n = 8)

Of the six informants who were sampled in the qualified family physician's clinic, only two of them considered themselves as having a family doctor, two considered themselves as having a regular doctor, and two considered themselves as having no regular doctor.

Interviews took place at a location of most convenience to the participant, and were conducted by an experienced qualitative researcher with a background in social and medical anthropology (JYS). All interviews were conducted in Cantonese. The interviewer is a native Cantonese speaker from Hong Kong but is also fluent in written and spoken English. The interviews followed a semi-structured format, and were free-flowing, and conversational in nature, using open questions to initiate discussion (see Additional file 2). Interviews lasted between 1 and 2 hours each, (shortest 57 minutes, longest 116 minutes).

### Data Analysis

Interviews were audio-recorded with the permission of participants and transcribed verbatim. The tapes were translated directly from Cantonese into English by bi-lingual transcribers, and all transcripts were checked by the bilingual research assistant to ensure accuracy of translation and transcription. The initial data analysis of the participants' transcripts was inductive, and no categories were specified in advance [9,10] A code book was kept to supplement the discussion generated from reading the transcripts. Based on careful reading and re-reading, the preliminary coding and categorization of the data was done independently by three experienced qualitative researchers (JYS, SWM, SMH) on different transcripts. Initial themes were identified and recorded by all three independently. The extent to which the categories and emergent theories identified by the researchers corresponded was discussed and the emerging codes and theories discussed and refined. Analysis proceeded through first to second level coding [11]. Regular meetings over the duration of the project allowed categorisation and classification, and the development of typologies and explanatory accounts to be pursued. This process utilised the constant comparative method - initially comparing data sets between individual transcripts, later comparing data with emergent hypotheses [9,10,12]. We were confident that we achieved data saturation in the present sample, and in later interviews no major new themes emerged from the data. Data analysis was manual and did not utilise any specific software.

The study was approved by the ethics committee of the Faculty of medicine, Chinese University of Hong Kong.

## Results

### Participant Characteristics

The characteristics of the participants interviewed are shown in Table 1. Those who considered themselves as having a family doctor were generally of higher socio-economic status (SES), and those with no regular doctor generally of lower SES, compared with the 'regular doctor but not a family doctor' group. For example, in the family doctor group, 6/10 (60%) had tertiary education compared with 4/10 (40%) in the 'regular doctor but not a family doctor' group and 0/8 (0%) in the no regular doctor group. In line with our aim of a maximum variation sample, there was a range of ages, genders, and types of chronic diseases represented in each group. However, there were fewer men in the no regular doctor group (2/8; 25%) compared with the other two groups (both 5/10; 50%). The mean age was also somewhat older in the no regular doctor group (65 years) than in the other two groups (47 years and 49 years for the family doctor group and regular doctor but not a family doctor groups).

### Knowledge and Understanding of the term 'Family Doctor'

Almost all participants had heard of the term 'family doctor' (except a few elderly participants of lower SES in the 'no family doctor; group). Participants' descriptions of what a family doctor is or does generally matched most of the accepted concepts of a family physician, i.e., care that is first contact, continuous, comprehensive, coordinated, and orientation to the patients' context (patient-centred) [13]. Unsurprisingly, this was especially true of those in the group with a family doctor but even those in the 'no family doctor' group generally at least knew of the concept.

"You will go to see that doctor for all diseases. And you can ask him questions if you have problems, and he will explain to you. Other ordinary western doctors will not do that. They will just talk to you around 3 sentences and you will have to leave then. They will not be in such a detailed manner. Family doctor is different. They are in more details. You can ask them questions on diseases and they will explain to you in detail." [23, no regular doctor]

Their information about family doctors came from a variety of sources, including the media. Several participants believed that a family doctor is a regular doctor to whom the whole family will go, and who has a close relationship with the family, almost like a 'family member'. Some felt that a family doctor would also be on-call 24 hours a day and be prepared to do home visits whenever requested.

#### Who should have a family doctor?

There were a diverse range of views on who should or could have a family doctor. Those with a family doctor felt that the family doctor model was appropriate, irrespective of age and type of condition. These participants were mainly of higher SES (see Table 1).

"I think I need a family doctor...I think that the health histories of mine and my ex-wife can influence our son, therefore I think if there's a doctor who can understand our health histories, all the better as he can have a clearer picture...I think this kind of doctor is very important, and there's such a need to have this kind of doctor, not only the doctor can have a clear picture on our health backgrounds, but we as a family can know how to prevent some diseases." [[7], has family doctor]

However, many participants who did not currently have a family doctor (either having a regular doctor who is not a family doctor or no regular doctor) and were mainly of lower SES than those with a family doctor (see Table 1), saw a family doctor as something of a 'luxury item' for the wealthy and not within the financial reach of the bulk of the population in Hong Kong.

"Seeing family doctors need money; and if you have money, then it's good. I cannot afford if I need to pay $1000 or $2000 every month. If I have money, of course I will go to find those family doctors. Even if I don't have any problems, I will go to them as well to ask questions. The most important thing is economy." [[17], has regular doctor]

#### Who can be a family doctor?

There was a majority view among participants (irrespective of whether they had a family doctor or not) that the family doctor model was only possible in the private sector and not feasible in the public healthcare sector. This was largely because of the perceived pressure on the public system including issues of access, lack of personal continuity, and time constraints.

There was very limited knowledge among participants (irrespective of whether they had a family doctor or not) about training or qualifications in family medicine, and the concept of a family doctor was not solely limited to general practitioners or family physicians. For two participants who classified themselves as having a family doctor, their family doctors were specialists in paediatrics and dermatology. There was no evidence that their understanding or expectations of a family doctor differed substantially from those with a specialist family physician as a family doctor. Most participants also believed that TCM practitioners have the potential to be family doctors.

"TCM practitioners are more willing to spend time listening to your problems. They will ask you many questions. From my experience, most TCM practitioners are really willing to spend time explaining about their prescription in details, and willing to understand our body status...they will explain your body situation in details, and they are willing to talk to you. I know the training of a TCM practitioner is much longer than the training of western medicine doctors, and some of them are doctoral graduates. Therefore, I think not only their qualification is better, but they know more about how to communicate with patients...I think TCM practitioners can become family doctors, because they also receive all-rounded training." [[18], with regular doctor]

### Views on the Current Primary Care System

#### The Public Primary Care system

The vast majority of participants were currently attending the public healthcare system (specialty outpatient clinics or general outpatient clinics) for their chronic diseases irrespective whether they had a family doctor or not. Reasons for this included issues of cost, consistency, informational continuity, prescription duration, quality, trust, access to specialists and allied health professionals (in-house referrals) and access to tests and investigations. For many (including those with a family doctor), they simply viewed the public system as the appropriate place to have their chronic disease managed.

"First, of course it is the economic concern. It's really very cheap. Also, if you need to have a surgery, or if you have any sudden changes in your disease, I think the equipment of public hospitals is better and more advance. But of course, the most important concern is finance. If you need to have more tests, then the fees will be more expensive in the private sector." [[13], with family doctor]

"It's better to go to public hospitals for chronic conditions, because hospitals are on a larger scale and so have better equipment like ECG and more senior doctors with better qualification and more experience. I trust hospitals more in this case. They have been following up my situation and so they don't need to redo the blood tests or other tests, because they have the records. If I switch to private doctors, then I will need to redo the blood tests and other tests as well as spending time to wait for the results. It's time consuming." [19, with a regular doctor]

There were thus many reasons for using the public system and advantages to 'getting into the system and staying there' with numerous disincentives to 'leaving the system'. This is not to say that participants were uncritical of the public healthcare system. Access, waiting times, a lack of interpersonal continuity, short consultations, and poor attitude of doctors were commonly cited as problems. It was also clear from participants' accounts that many factors conspired to keep them in the public healthcare system, including recommendations by their private doctors, and ongoing internal referrals with no effective linkage to the private sector.

#### The Private Primary Care System

Private primary care doctors were generally regarded as being mainly for acute illnesses, rather than for chronic disease management. Participants expressed a general lack of trust in the private sector, particularly among those who did not have a family doctor. Reasons for this included cost of consultations and prescriptions, ineffectiveness of treatments, concerns about lack of training and knowledge, and suspicions about being overcharged in terms of unnecessary drugs and investigations. Many participants voiced concerns about the government's planned healthcare reforms, and possible public-private partnerships between the HA and the private GP sector. Cost was a major concern for many, especially those on lower incomes, but issues of quality assurance also figured highly.

"But I am worried about the quality. I am afraid that these family doctors are not as knowledgeable as the public doctors. You know, we have many diseases, so it's more troublesome. If you just have a cold and flu, then of course it's easy and every doctor can treat you. But if you say heart problems, I am afraid that family doctors are not capable and they may not have such expertise. I still think that public doctors are better to follow my chronic diseases." [25, no regular doctor]

### Barriers and Incentives to Adopting the Family Doctor Model

There were five main themes identified; cost, perceived need, choice, relationships, and quality issues.

#### Cost

For many interviewed cost was a major barrier, especially for those who did not have a family doctor, which generally reflected income level and SES; those on higher incomes were less concerned about costs personally (for themselves and their family) whereas those on lower incomes were greatly concerned.

"Many people are still struggling on how they can pay the consultation fees, so how can they have the ability to talk about family doctors?" [20, with regular doctor]

"I think family doctor is a very extravagant thing. I think family doctors will be very expensive...It will cost you several hundred for a single visit. It's really a waste. I think it's really a waste to spend several hundred dollars on seeing doctors for the things that you can do by yourself. We are in lower class and several hundred is too expensive for us...." [25, no regular doctor]

However, the relationship with cost was not entirely straightforward. There was a view expressed by some participants that 'good things can't be cheap', i.e., high quality family medicine *should *be expensive:

"I just think that if a family doctor has good expertise, then he should not be cheap. Cheap things should not be good, as I have experienced this before." [25, no regular doctor]

#### Perceived need

A second barrier to the adoption of the family doctor model was the perception by many participants of the lack of need. Many of those without a family doctor simply saw having one as something unnecessary, irrespective of financial issues:

"It's not a money problem. Because I don't think I have the need to have one. Not every family needs a family doctor. If you and your children are healthy, what's the need of having a family doctor? If your children have many diseases, then you may need to have one... It's not necessary for one to have a family doctor if he just merely suffers from a cold and flu occasionally." [19, with regular doctor]

"There's no such need at the moment. I don't have too much sickness... I think going to hospital is good enough... There is no such need. I don't need a [family] doctor to follow my case closely." [28, with no regular doctor]

Conversely, some others did perceive the need for family doctors, which was related to perceptions of risk and concurrent diseases and to a large extent current or past experience of having a family doctor. For some, the incentive to have a family doctor came from positive experiences of family doctors overseas, in countries with well-developed primary care systems.

"I think I need a family doctor. Migrating to Canada was really the changing point. My ex-wife and my son also saw that family doctor in Canada...therefore after coming back to Hong Kong, I also hope that I can have the same doctor to follow my whole family's health, which I think it is good to my son and to myself....I think this kind of doctor is very important, and there's such a need to have this kind of doctor, not only the doctor can have a clear picture on our health backgrounds, but we as a family can know how to prevent some diseases." [[7], with a family doctor, the first experience of family doctor was in Canada]

#### Choice

The right to choose a doctor was a common theme which was related exclusively to the private sector. Participants strongly defended their right to choose a doctor (or doctors), in order to find the 'right match'. 'Doctor shopping' was regarded as a way to assert choice in order to find a good doctor. Thus a potential barrier to the adoption of the family doctor model was the concern that the government might limit choice by imposing restrictions; although financial incentives or subsidies on the one hand were seen as an incentive to moving to a family doctor model, many also wanted reassurance that the 'right to choose' would not be diminished.

"Family doctors should be something like options and choice, and should not be a mandatory thing, because many people are very conservative and don't want other people know too much about them, even though they are doctors." [[10], with family doctor].

"If the government assigns a family doctor to a patient, then I cannot know whether this doctor really suits me." [[13], with a TCM practitioner as family doctor]

Interestingly, most felt that this 'right to choose' simply did not apply to the public healthcare system. Although in principle they would prefer to be able to see a doctor of their choice in the public system, and be able to form a long-term relationship with that doctor, in practice many felt this was simply impossible.

#### Doctor-patient relationship

A related barrier to adopting the family doctor model was the issue of the doctor-patient relationship. Many participants had concerns about the attitudes of doctors in Hong Kong. Many quoted examples of commonly being rushed in consultations, with little time to ask questions, gain information, and so on:

"It's really rare to see a doctor who can spend 5 minutes on a patient, and I think its standard to finish a patient in one minute. Probably, they still haven't seen your face but have started to type your record and prescription. They will then ask you whether you need a sick-leave certificate. That's it. Everyone is in a rush. Patients are in a rush, and the doctors are in a rush. Therefore, it's really difficult to have a family doctor in Hong Kong." [20, has regular doctor]

"It is not easy to get a good doctor who is willing to spend time and have the patience to listen to you. I don't know where I can find such a doctor. Most doctors are very busy in 'rushing cases'." [[18], has regular doctor]

Conversely, an important incentive to having a family doctor was the possibility of forming an enduring therapeutic relationship. There were numerous potential advantages associated with this, such as effectiveness, efficiency, holistic support, empathy, respect, trust, confidence, health promotion and self-care support. However, many felt that a therapeutic relationship with a family doctor would take a long time to develop; that is the relationship had to be developed and nurtured over a period of years. This was irrespective of the doctors training, qualifications, or certificates. Respect and trust had to be earned through contact and experience, and the patient's judgement of the doctor's skills by their own personal evaluation of honesty, integrity, and effectiveness of care.

Although participants generally viewed the long-term therapeutic relationship with a doctor as a positive factor, one participant felt that 'being too familiar' with a doctor could be a barrier.

"Maybe I am too familiar with him and have seen him for a long time, so I don't dare to tell him, or I don't feel comfortable psychologically to tell him the truth if his medication fails. It's quite contradictory. Of course it's good to have a doctor whom you have seen for a long time. But if his medication fails, I don't dare to tell him honestly... I don't dare to tell him, because he may think that I feel suspicious with his medication, though I have seen him for a long time. I am afraid that the doctor will have such feeling. Some doctors are better because they will really tell you that you have to go back to switch to another medication if this medication cannot work for you. You will feel less embarrassed to go back. But if the doctor told you that "it's okay for you to take this medication", then how can you go back and tell him the medication fails? I think this is really embarrassing." [27, no regular doctor]

#### Quality issues

A further theme which acted as a barrier to having a family doctor was the issue of quality. Many participants were concerned that private family doctors were not adequately trained or skilled to deal with chronic diseases. Some felt that only specialists could look after specific chronic conditions, and therefore family doctors had to be specialists in the patient's particular disease.

"I will find a specialist as my family doctor for my heart problems and not just a GP... I do not expect there's a doctor who can follow all my diseases - it seems that he's not a specialist al all; I just expect that I can follow the same doctor in internal medicine, and the same doctor in ENT [ear, nose and throat]...I think there is no single doctor can be all-rounded. An ENT specialist will just have titles on ENT. They do not have cardiology titles. I think this is a barrier. Just like I have heart and ENT diseases; but others may have even more different types of diseases which may involve many specialties, then how can you find a doctor who can treat all these diseases?" [[13], TCM as family doctor]

"I think for specialty problems, family doctors are incapable in dealing with them... Of course only the specialists can deal with specialty problems. Family doctors cannot, so of course it's better to search the specialist by myself if I think it's a specialty problem... Family doctors only deal with minor diseases, they are not specialists. If I need specialist treatment, I still have to see the specialists no matter how far they are." [26, no regular doctor]

Qualifications and certificates were rarely used by participants as criteria on which to judge if a doctor was suitably qualified to deal with chronic diseases. Indeed the issue of trust was not simply related to knowledge, but was also intimately related to perceptions of the doctors' ethics and values. Even for those participants who believed that further training for family doctors should be required, they felt the training should focus on holistic and humanistic skills as much as medical skills.

"But training can just teach you about the skills; whether you can do well as a family doctor depends much on your personality. Medical skills are not important for family doctors, but what's more important is that whether they can motivate a patient to tell the real health situation to them, and so they can help you thinking about the possibilities to treat your problem. It's really based on trust. The main function of family doctors is not for treatment, but he is the one that you can trust and he can give you medical advice. I think what the subject or training can teach mainly concerns about the skills, but it is still impossible for them to become family doctors if the environment does not allow." [20, with regular doctor].

Finally, some participants said they did not know how to find a family doctor in Hong Kong.

"In Hong Kong, I just have the impression that you don't know where you can go when you are sick - the only place that you can go is the doctor whom is convenient to you. Otherwise, you can only go to public clinics. I don't know if Hong Kong has family doctors. Maybe there are some family doctors, but I cannot know, and I don't know any sources that I can know whether there are family doctors here... I do not have the information of how to get a family doctor from any people. I can ask my friends for recommendation about a good acupuncturist, but no friends have ever recommended me about a family doctor, because people do not have the concept of family doctor." [21, with a regular doctor, past experience of family doctor in Australia].

### Summary of similarities and differences between views of participants with a family doctor and those with no family doctor

In summary, there were similarities and differences in the perceptions, knowledge, and understandings of the family doctor model between the participants who had experience of a family doctor (family doctor group) and those with no experience (regular doctor/no regular doctor groups) as shown in table 2. In general, most participants had a reasonably accurate knowledge of the family doctor model, participants in both groups equated it with the private sector, all participants felt TCM practitioner had the potential to be family doctors, and participants in both groups generally felt that chronic disease management was best done by the public system.

**Table 2:** Differences and similarities between informants with and without family doctors

	Family doctor group (n = 10)	No family doctor group (n = 18)
Knowledge of family doctor model of care	√√√	√√
Family doctor can only be in private sector	√√√	√√√
Family doctor can be TCM doctor	√√√	√√√
Chronic disease management best done in public sector	√√√	√√√
Family doctor offers holistic care and therapeutic relationship	√√√	√
Family doctor is essential to health needs	√√√	XXX
Family doctor is costly	√	√√√

Choice and quality key issues	√√√	√√√

Those with a family doctor valued the holistic approach and the therapeutic relationship that developed over time, and felt that they and their families needed a family doctor. Those without a family doctor saw the potential benefit of such a relationship, but felt that having a family doctor was a 'luxury' rather than a 'need'. Cost was a concern, especially for those without a family doctor (who were of lower SES), as was trust in quality of care. All groups valued the preservation of choice which came with the private sector. These views on family doctors were not obviously related to gender or age, and therefore the fact that those in the 'no regular doctor' group were somewhat older on average and mainly female (Table 1) did not seem to account for these findings. The main factor associated with having a family doctor or not (and hence the specific views related to that) was SES, which was generally higher in those with a family doctor (Table 1).

## Discussion

The aim of the present study was to explore the incentives and barriers to adopting the family doctor model in Hong Kong from the viewpoint of participants with chronic disease by means of in-depth semi-structured interviews. That most participants had some knowledge of the concept of a family doctor is in agreement with a recent survey of over 1,000 members of the public conducted by the Hong Kong College of Family Physicians which found that over 90% of respondents had heard of the term family doctor [14]. The survey also showed that cost was the most important issue influencing choice of service and that in terms of dealing with chronic illness, only a minority felt that private doctors were capable of doing this, again in line with our own findings.

In the present study it is noteworthy that the family doctor model was generally equated with the private sector and that family doctors were regarded as something of 'a luxury item for the rich' by those who did not have one. Virtually all participants (irrespective of whether they had a family doctor nor not) attended the public healthcare system for ongoing management of their chronic diseases. There were many reasons for this (cost, consistency, informational continuity, prescription duration, quality, trust, access to specialists, allied healthcare professionals, tests and investigations) and many forces seemed to conspire to keep patients within the public healthcare system, both from within the system and from without. However, for most participants, the public healthcare sector was simply regarded as the appropriate setting for chronic disease management. Thus 'shifting the balance of care' from the public healthcare system to the private system, or even moving to a more 'shared-care' system between public and private providers, as suggested in the recent consultation document on health care reforms in Hong Kong [6] is unlikely to be a straightforward matter.

An additional problem in the context of chronic disease management was the fact that private primary care was mainly seen as dealing with acute minor illness, or in the case of TCM as a general health 'tonic'. This is supported by the findings of Dickinson [15] which showed that people tend to consult private doctors for minor illnesses. It is of interest that the majority of our participants felt that TCM practitioners had the potential to become family doctors. The reasons varied but most cited the therapeutic relationship, the cultural 'fit' of TCM, and the relatively detailed explanations received when compared with western doctors. Wong et al. [16] also contended that TCM practitioners gave more detailed explanation to patients. However, integration of TCM with western medicine remains problematic in Hong Kong, and issues of safety and effectiveness remain unanswered.

Gauld and Gould [17] reported that around three-quarters of all patients were interested in knowing more about their diseases, and the participants in the present study generally expressed a keen interest in knowing more about their health problems. Participants tended, in this respect, not to enquire and seek explanations from the doctors in public clinics about their diseases due to the short time available in the consultation. Possibly when patients are paying they feel more able to assert their 'purchasing power' and demand more from consultations. Such differences in expectations would be consistent with Gauld's and Gould's previous findings[17].

In terms of barriers to adopting the family doctor model, cost was a major concern for many, but issues of quality, need, and choice were also important barriers to adopting the family doctor model. Incentives included the perceived benefits of a long-term therapeutic relationship with a family doctor, and the possibility of government financial subsidies. Similar to the findings of Lester et al. [18], most participants in our study saw the potential benefits of having a continuous doctor-patient relationship and seeing the same doctor at each primary care visit. The fact that participants knew little about the Hong Kong College of Family Physicians, nor how to find a qualified family doctor possibly reflects both the limited number of fully qualified family physicians in Hong Kong, and the lack of published information available to the public.

### Strengths and limitations

The present study adopted qualitative methodology and such an approach is especially useful in elucidating issues of context, depth, detail, and content [7]. The choice of qualitative methods was, we feel, an appropriate one as we sought to describe what existed 'out there' in the study sample, including motivations, experiences and contexts and the reasons for and associations between them [8]. Many of the underlying reasons and beliefs elicited in the present study simply could not have been identified by quantitative research methods.

Sample size in qualitative research is not set in advance, but depends on the context of the study and when saturation is reached in terms of new themes [7,8]. Large numbers are not required. In the present study we noted saturation of themes before the last interviews were completed, and thus are satisfied that our sample size was sufficient for the purposes of the study. However, it should be noted that the characteristics of the different groups did differ somewhat; those with a family doctor were generally of higher SES, and in the 'no regular doctor' group there were more women and the ages tended to be older. We cannot therefore be sure that these differences did not 'contaminate' our findings, though we looked for and did not find any major effects of age and gender on the themes uncovered.

We recruited respondents from two sources; a linked survey carried out by Lam and colleagues (22 respondents) and an additional 6 respondents from the clinic of a family medicine specialist, in order to try to discern if the views expressed by those who considered themselves as having a family doctor (of unknown qualification) were similar or different to those attending a known and fully qualified family medicine specialist. We had assumed that all 6 would consider themselves as having a family doctor and were also surprised that only 2 out of these 6 informants did. We cannot fully explain why this was, but perhaps reflects the Hong Kong private primary care system and the phenomenon of 'doctor shopping'. It suggests that in the Hong Kong private sector having a qualification of family medicine specialist does not necessarily influence informants' perceptions of having a family doctor.

### Implications for policy and practice

If the findings of this qualitative study are representative of this patient group, the successful implementation of a comprehensive family doctor system for chronic disease management in Hong Kong seems unlikely unless the multiple, complex barriers identified are addressed with a systems approach. The cost and trust barriers have important implications for policy makers with respect to the proposed health care reforms in Hong Kong which aim to improve the integration of the private and public primary care systems and to shift the balance of care for chronic disease management from secondary to primary care. The public has yet to be convinced of the competence of family doctors in chronic disease management. Potential ways forward for policy and decision makers to help overcome these barriers could include:

1. Promotion of the family doctor model in Hong Kong by a media campaign aiming to promote a positive (but realistic) image of family doctors and their abilities to manage chronic as well as acute illnesses

2. Financial incentives to enable patients with chronic diseases to be able to afford having a family doctor within the private sector

3. Financial incentives for family doctors to promote continuity of care

4. Verifiable safety and quality assurances so that patients can trust family doctors in the private sector to manage their chronic diseases appropriately

5. A requirement for family doctors to demonstrate competence and up-to-date knowledge of evidence-based management of common chronic diseases and patient-centred communication skills

6. Availability of high quality training for family doctors in chronic disease management

7. Promotion of the family doctor model in public healthcare sector and better communication and integration between public and private sector

## Conclusions

According to the views of patients with chronic diseases in this study, the introduction of a family doctor model in Hong Kong will face major barriers. Unless the attitudes and perceptions described above are addressed, the effective implementation of a comprehensive family doctor system for chronic disease management in Hong Kong seems likely to fail, if the findings from this study are generalisable to the wider population.

## Competing interests

The authors declare that they have no competing interests.

## Authors' contributions

SWM conceived and designed the study and was involved in analysis and interpretation of the data, drafting and revising the manuscript. JYS helped with design, collected the data, carried out the analysis, and helped draft the manuscript. CLK, TPL, SMH, YYCL and SMG assisted in the conception and design of the study, commented on the analysis and interpretation, and helped revise sections the manuscript. SMH also contributed to the analysis in depth, working closely with SWM and JYS. All authors contributed intellectually at all stages and all gave final approval.

## Pre-publication history

The pre-publication history for this paper can be accessed here:

http://www.biomedcentral.com/1471-2296/11/46/prepub

## Supplementary Material

Additional File 1 **Questionnaire used to recruit patients**. A copy of the survey tool used in the recruitment of patients into the study. Click here for file

Additional File 2 **Interview guide for the semi-structured interviews**. Details of the questions used by the interviewer in the qualitative interviews. Click here for file
