# Supplementary material for: A qualitative study of the views of patients with long-term conditions on family doctors in Hong Kong
Source: BMC Fam Pract. 2010 Jun 4;11:46. doi: 10.1186/1471-2296-11-46 (PMC2889885; doi:10.1186/1471-2296-11-46)
Supplement: Additional File 1 — Questionnaire used to recruit patients. A copy of the survey tool used in the recruitment of patients into the study. [file 1471-2296-11-46-S1.DOC]

# Additional files

### Additional file 1 – Questionnaire used to recruit patients

| **Morbidity & Usual Utilization Pattern** | | | | | |
| --- | --- | --- | --- | --- | --- |
| 2. | Do you have a regular primary care doctor? A primary care doctor is a doctor whom you would first consult when you need to. | 1. Yes (please proceed to answer 3a) 2. No (please proceed to answer 3b) 3. Not sure (please proceed to answer 3b) | | | |
| 3 | a. Is your regular primary care doctor a family doctor? A family doctor is a doctor whom you would consult for all types of health problems. | 1. Yes 2. No, he/she is other types of general practitioners 3. No, he/she is a specialist in ______________ 4. No, he/she is a traditional Chinese medicine practitioner 5. Not sure | | | |
|  | b. Do you have a family doctor? | 1. Yes, a general practitioner  2. No  3. Not sure | | | |
| 4. | Think about a typical primary care consultation that you had. How long did it last? | _____________ minutes. (-1 for not sure) | | | |
|  |  | | 1.Yes | 2.No | 3.Not sure |
| 5. | **Have you ever been diagnosed by a Western doctor to have the following diseases for more than four weeks?** | |  |  |  |
|  | - 1. hypertension | |  |  |  |
|  | 1. diabetes mellitus | |  |  |  |
|  | 1. any heart disease | |  |  |  |
|  | 1. stroke | |  |  |  |
|  | 1. asthma, emphysema, chronic bronchitis or other chronic lung diseases | |  |  |  |
|  | 1. arthritis or other chronic joint problems | |  |  |  |
