# Supplementary material for: A qualitative study of the views of patients with long-term conditions on family doctors in Hong Kong
Source: BMC Fam Pract. 2010 Jun 4;11:46. doi: 10.1186/1471-2296-11-46 (PMC2889885; doi:10.1186/1471-2296-11-46)
Supplement: Additional File 2 — Interview guide for the semi-structured interviews. Details of the questions used by the interviewer in the qualitative interviews. [file 1471-2296-11-46-S2.DOC]

### Additional file 2 – Interview Question Guide for the Semi-Structured Interviews

1. Explore the views of patients with long-term conditions on primary care as it currently exists
2. Examine their knowledge and understanding of the family doctor model
3. Delineate the incentives and barriers to adopting the family doctor model
4. Elucidate their attitudes towards health, self-care, and the role of different primary care providers

A.

1. How will you define health? Do you think that you are healthy? Why do you think so?
2. Can you tell me whether you are also suffering from other chronic conditions other than the physical problems that you are suffering now?
   - if yes, can you tell me more about what kinds of chronic conditions that you are suffering?
3. Where do you go first when you feel you are concerned about a health problem? (does this differ from an acute illness compared with the long-term illnesses?) And why? Is this doctor the one that you go most often?
4. Do you follow the same doctor / or see another different doctor for those chronic conditions that you have just mentioned? (🡪 specialty fragmentation, doctor shopping) And why do you have such choice? / Why did you first go to that doctor for your chronic conditions? / What influences your choice (Explore in detail)
5. (If informant sees another doctor for other chronic conditions)
   - Is that doctor a specialist / GP?
   - Under what occasions that you will come to see this doctor?
6. If you believe you have some acute diseases, then will you go to the same doctor or to another different doctor? And why?

B.

1. Have you ever heard about “family doctor”? If yes, can you tell me your understandings about “family doctor”? / What do you understand by the term ‘family doctor’? (first contact, generalist, continuous, comprehensive – curative + rehabilitative + supportive + health promotion + disease prevention, orientation to patient’s context – personal and social context in the treatment, coordinated care as gatekeeper of specialist care)
2. Then do you have a “family doctor”? (can include family physicians, GPs, TCM practitioners)
   - If yes, is it the doctor that you go most often? How long do you have him / her as family doctor?
   - Why do you perceive him / her and want to have him / or her as your family doctor?
   - Why do you want to have a family doctor? What are your concerns for having a family doctor?
   - If no family doctor, do you have a particular doctor that you go most often for health problems?
   - Do you go to different doctors for different types of health problems? If yes, how do you determine which doctor you should go?
   - Why don’t you follow the same doctor for your health problems?

C.

9. In your perception, who should be the family doctor? What qualities do you believe that a family doctor should possess? (a fully trained family physician, untrained GP, GP with some family medicine training, TCM practitioner, etc.)

10. What would be the main incentives to having a family doctor?

11. What will be the main barriers to having a family doctor?

D.

12. Besides consulting a doctor, do you take any actions for taking care of your chronic conditions? (such as exercises, taichi / *qigong*, TCM and any other CAMs)

- - if yes, how and where do you learn these actions / approaches to take care of your chronic conditions? (such as family, relatives, friends…)
  - what role do different care providers play in this, if any?
  - when did you start adopting these approaches when you first discovered your chronic conditions?
  - Why do you choose adopting these alternative approaches in dealing with your chronic conditions? What roles do they play in your chronic conditions?
  - Can you experience any differences after adopting these approaches? If yes, what are the differences, good or bad?
  - Are these self-care approaches important to you?

Note: in semi-structured interviews, questions for individual participants will be slightly different as follow-up questions will have to be asked in response to different participants. The above is the key questions that covered all participants.
